# Clinical and animal research findings in pycnodysostosis and gene mutations of cathepsin K from 1996 to 2011

**DOI:** 10.1186/1750-1172-6-20

**Published:** 2011-05-10

**Authors:** Yang Xue, Tao Cai, Songtao Shi, Weiguang Wang, Yanli Zhang, Tianqiu Mao, Xiaohong Duan

**Affiliations:** 1 Department of Oral and Maxillofacial Surgery, School of Stomatology, the Fourth Military Medical University, 145 West Changle Road, Xi'an 710032, P. R. China; 2 Department of Oral Biology, School of Stomatology, the Fourth Military Medical University, 145 West Changle Road, Xi'an 710032, P. R. China; 3 Oral Infection and Immunity Branch, National Institute of Dental and Craniofacial Research, National Institutes of Health, Bethesda, Maryland 20892, USA; 4 Center for Craniofacial Molecular Biology, University of Southern California School of Dentistry, Los Angeles, California 90033, USA; 5 Center of Cardiovascular Surgery, Xijing Hospital, the Fourth Military Medical University, 145 West Changle Road, Xi'an 710032, P. R. China

**Keywords:** cathepsin K, pycnodysostosis, osteoclast, bone, oral deformities

## Abstract

Cathepsin K (CTSK) is a member of the papain-like cysteine protease family. Mutations in the *CTSK *gene cause a rare autosomal recessive bone disorder called pycnodysostosis (OMIM 265800). In order to follow the advances in the research about CTSK and pycnodysostosis, we performed a literature retrospective study of 159 pycnodysostosis patients reported since 1996 and focused on the genetic characteristics of *CTSK *mutations and/or the clinical phenotypes of pycnodysostosis. Thirty three different *CTSK *mutations have been found in 59 unrelated pycnodysostosis families. Of the 59 families, 37.29% are from Europe and 30.51% are from Asia. A total of 69.70% of the mutations were identified in the mature domain of *CTSK*, 24.24% in the proregion, and 6.06% in the preregion. The hot mutation spots are found in exons 6 and 7. *CTSK *mutations result in total loss or inactivity of the CTSK protein, which causes abnormal degradation of bone matrix proteins such as type I collagen. Skeletal abnormalities, including short stature, an increase in bone density with pathologic fractures, and open fontanels and sutures, are the typical phenotypes of pycnodysostosis. Research on *Ctsk*^-/- ^mouse models was also reviewed here to elucidate the biological function of *Ctsk *and the mechanism of pycnodysostosis. New evidence suggests that *Ctsk *plays an important role in the immune system and may serve as a valid therapeutic target in the future treatment of pycnodysostosis.

## Introduction

Pycnodysostosis (OMIM 265800) is a rare autosomal recessive bone disorder resulting from osteoclast dysfunction [1-4]. The first case of pycnodysostosis was described in 1923 by Montanari; however, Maroteaux and Lamy defined the typical features of pycnodysostosis (Greek: pycnos = dense; dys = defective; osteon = bone) in 1962. Thus, it is also known as Maroteaux-Lamy syndrome. This disorder is also called Toulouse-Lautrec syndrome after the famous French artist Henri de Toulouse-Lautrec, who was thought to be afflicted with the disease [2,3,5]. Less than 200 cases have been reported worldwide since 1962 [1]. The prevalence of pycnodysostosis is estimated to be 1 to 1.7 per million with equal sex distribution [4-7]. The typical features of pycnodysostosis include short stature, an increase in the bone density of long bones, pathological fractures with poor healing, stubby hands and feet with dystrophic nails, unossified fontanels, and an obtuse mandibular angle [5,8-10].

The candidate gene for pycnodysostosis was mapped to human chromosome 1q21 by genetic linkage analysis, and was subsequently identified as coding for cathepsin K (*CTSK*, MIM# 601105) by a positional cloning strategy in 1996 [11-13]. The *CTSK *gene spans approximately 12 kb (*GenBank acc. no.*, NC_000001.10) and contains 8 exons (*GenBank acc. no.*, NM_000396.2). The codon for the translation initiator methionine (Met1) is located in exon 2, whereas the termination codon is located in exon 8. The CTSK protein, highly similar to cathepsins S and L, is a member of the papain-like cysteine protease family. Like most papain-like cysteine proteases, CTSK consists of 329 amino acids (*GenBank acc. no.*, NP_000387.1), including a 15-amino acid preregion, a 99-amino acid proregion, and a 215-amino acid mature active enzyme [14]. CTSK is synthesized as an inactive precursor protein and requires removal of its N-terminal proregion for activation. This autocatalytic process occurs under a low-pH environment [3].

To date, it is difficult to find a full description of the characteristics of specific gene mutations and clinical manifestations of pycnodysostosis in the literature. In this study, we analyzed the reported *CTSK *mutations and summarized the typical clinical features of pycnodysostosis from 159 reported patients and some animal research findings in *Ctsk *gene knockout mice (*Ctsk*^*-/- *^mice). Early reports of *CTSK *mutations were renamed in this study with the first base in the *CTSK *gene (*GenBank acc. no.*, NC_000001.10) as the +1 position in genomic DNA, and the A of the ATG-translation initiation codon as nucleotide +1 in cDNA (*GenBank acc. no.*, NM_000396.2).

### Structure of CTSK

CTSK is also called lysosomal cysteine cathepsin K because it contains a cysteine in its active site and functions mainly in lysosomes [15]. Like most lysosomal cysteine proteases, CTSK is synthesized as an inactive proenzyme [3,16]. The preregion plays a critical role in targeting the protein to the endoplasmic reticulum and translocating the protein across the membrane [17-19]. While the proregion plays a role in protein folding and intracellular trafficking, it can also inhibit protease function until the proenzyme reaches the lysosome [20]. The proregion contains a conserved N-glycosylation site (Asn103), which is supposed to facilitate lysosomal trafficking via the mannose 6-phosphate receptor pathway [11,14,20]. The proenzyme requires removal of its N-terminal proregion for activation [16]. This process has been proven to be autocatalytic in lysosomes at a pH of 4 [3,21]. CTSK consists of 2 domains folded together, resulting in a V-shaped configuration [22,23]. The catalytic triad, consisting of Cys139, His276, and Asn296 in the active sites, localizes at the bottom of the V cleft [14,15,24,25].

### Distribution of CTSK

CTSK is highly expressed in osteoclasts and has lower expression levels in the heart, lung, skeletal muscle, colon, ovary, and placenta [3,5,20]. Additionally, *CTSK *mRNA was detected in macrophages and bone marrow-derived dendritic cells, but was barely detected in nonadherent bone marrow cells or splenic T cells [26,27].

### Function of CTSK

#### In osteoclasts

CTSK, which is critical for osteoclast-mediated bone resorption, is highly expressed in osteoclasts. In osteoclasts, CTSK is responsible for the degradation of bone matrix proteins, such as type I collagen, osteopontin, and osteonectin [3,5,20]. A tightly sealed resorption lacuna between the osteoclast and the bone is called an extracellular lysosome. Dissolution of the inorganic matrix and degradation of the organic matrix occur in the extracellular lysosome under low pH conditions [28,29]. In mature osteoclasts, CTSK is synthesized as an inactive proenzyme and cleaved by autoproteolysis to produce the active form of the protein. This active form is then secreted into the extracellular lysosome [30-32], where it degrades bone matrix proteins, particularly type I collagen, which constitutes 95% of the organic bone matrix [3,5,15,20,33]. CTSK deficiency does not affect the function of osteoclast-mediated extracellular acidification [34]. *Ctsk *mutations were found to impair the ability of osteoclasts to degrade collagen rather than demineralize the extracellular matrix.

On the other hand, CTSK may also act as a potential regulator of apoptosis and senescence, controlling osteoclast numbers *in vivo *[34]. Thus, impairment of CTSK-mediated osteoclast apoptosis/senescence may also be responsible for the higher number of osteoclasts found in *Ctsk*^*-/- *^mice [34].

#### In immunocytes

A pycnodysostosis patient with normal immune status was reported in 1999 [35]. However, impaired killing activity of monocytes with normal phagocytic capacity and decreased levels of IL-1 secretion were reported in pycnodysostosis patients in an earlier study [36].

In an early animal study, abnormities of histological morphology or cellularity were found neither in the thymus nor in the levels of B and T lymphocytes in peripheral blood [37]. Fluorescence-activated cell sorter analysis showed no difference in the lymphocyte markers (CD4, CD8, CD3, B220, IgM, and IgD) between *Ctsk*^-/- ^and wild-type mice. Immunophenotype analysis of the cell types in the bone marrow revealed a significant decrease in the absolute cell number of all subtypes, even though the percentage of each subtype in the entire population was unchanged [37].

Recently, Ctsk was found to function in the endosomes of dendritic cells. Pharmacological inhibition or targeted disruption of *Ctsk *led to defective Toll-like receptor 9 signaling in dendritic cells when stimulated with cytosine-phosphate- guanine, but not when stimulated with lipopolysaccharide or peptidoglycan. It was shown that *Ctsk *is indispensable for differentiation of dendritic cells, but not required for antigen uptake, processing, or presentation by dendritic cells. The same study also indicated that the ability of dendritic cells to induce T helper 17 (Th17) cells was markedly inhibited by *Ctsk *inactivation, which may be caused by a reduction in the expression of Th17 cell-related cytokines, such as IL-6 and IL-23, by dendritic cells. Furthermore, *Ctsk*^*-/- *^mice were resistant to experimental autoimmune encephalomyelitis, in which Th17 cells are involved [26]. These results suggest that *Ctsk *plays an important role in the immune system and may serve as a valid therapeutic target in autoimmune diseases.

Nevertheless, it remains to be determined whether CTSK plays a pathogenic role in the human immune system or in autoimmune/inflammatory diseases.

#### In other cells and tissues

New evidence suggests that CTSK is involved in extracellular matrix remodeling in organs such as the lung, thyroid, and skin, and plays a critical role in the development and progression of cardiovascular disease [38]. Extensive destruction of elastin and collagen caused by overexpression of cathepsins K and S has been related to the damage and inflammation of arterial wall, resulting in atherogenesis [15,38-41].

### Variants in the CTSK gene

**Figure 1:**
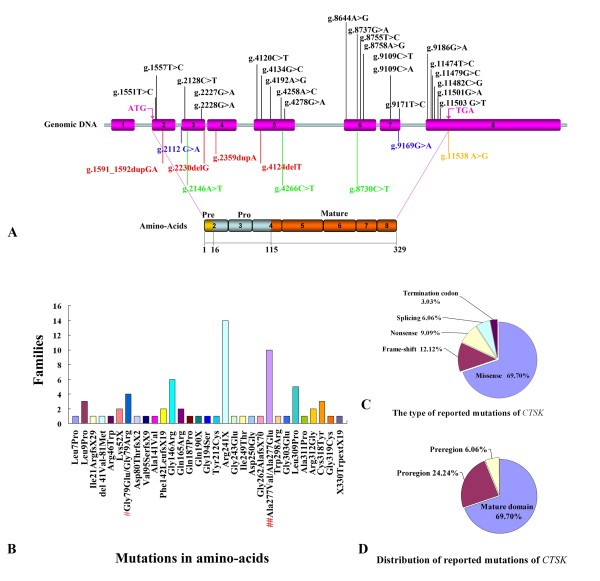
**Reported mutations of *CTSK***. (A) Distribution of the *CTSK *gene and polypeptide mutations. The genomic structure of the *CTSK *gene with 8 exons (purple boxes numbered 1-8) is shown in the top half. The bottom half illustrates the schematic representation of the polypeptide comprising a 15-amino acid preregion (yellow box), a 99-residue proregion (light blue boxes), and a 215-amino acid mature domain (orange boxes). A total of 23 missense mutations (black type) are represented at the top of the gene diagram, while frame-shift mutations (red type), nonsense mutations (light green type), splicing mutations (blue type), and termination codon mutations (yellow type) are at the bottom. (B) Frequency of different mutations. The height of each bar represents the number of afflicted families. #: Both mutations in the Glu70 residue. ##: Both mutations in the Ala277 residue. (C) The type of reported *CTSK *mutations. The mutations reported in pycnodysostosis patients consist of 23 missense mutations, 4 frame-shift mutations, 3 nonsense mutations, 2 splicing mutations, and 1 termination codon mutation. (D) Distribution of reported *CTSK *mutations. A total of 69.70% of the mutations occurred in the mature domain, 24.24% in the proregion, and 6.06% in the preregion.

Thirty three different mutations have been reported in 59 pycnodysostosis families [1,3,8,9,14,17,20,42-54] (Table 1, Figure 1A). The Arg241 in exon 6 and Ala277 located in CpG dinucleotides in exon 7 are two mutational hot spots for pycnodysostosis (Figure 1B). Various mutations have been reported in pycnodysostosis patients, including 23 missense mutations (69.70%), 4 frame-shift mutations (12.12%), 3 nonsense mutations (9.09%), 2 splicing mutations (6.06%), and 1 termination codon mutation (3.03%) (Figure 1C). A total of 69.70% of the mutations occur in the mature domain of *CTSK*, 24.24% in the proregion, and 6.06% in the preregion (Figure 1D).

**Table 1:** Mutations in the *CTSK *gene causing pycnodysostosis

Location in DNA sequence	Genomic DNA sequence variants	Coding DNA sequence variants	Effect on amino acid	Location in protein sequence	First description
**Missense**					
Exon 2	g.1551T > C	c.20T > C	p.Leu7Pro	Pre	Donnarumma, et al., 2007
Exon 2	g.1557T > C	c.26T > C	p.Leu9Pro	Pre	Nishi, et al., 1999
Exon 3	g.2128C > T	c.136C > T	p.Arg46Trp	Pro	Schilling, et al., 2007
Exon 3	g.2227G > A	c.235G > A	p.Gly79Arg	Pro	Fratzl-Zelman, et al., 2004
Exon 3	g.2228G > A	c.236G > A	p.Gly79Glu	Pro	Hou, et al., 1999
Exon 5	g.4120C > T	c.422C > T	p.Ala141Val	Mature	Chavassieux, et al., 2008
Exon 5	g.4134G > C	c.436G > C	p.Gly146Arg	Mature	Gelb, et al., 1996
Exon 5	g.4192A > G	c.494A > G	p.Gln165Arg	Mature	Donnarumma, et al., 2007
Exon 5	g.4258A > C	c.560A > C	p.Gln187Pro	Mature	Li, et al., 2009
Exon 5	g.4278G > A	c.580G > A	p.Gly194Ser	Mature	Donnarumma, et al., 2007
Exon 6	g.8644A > G	c.635A > G	p.Tyr212Cys	Mature	Hou, et al., 1999
Exon 6	g.8737 G > A	c.728G > A	p.Gly243Glu	Mature	Khan et al., 2010
Exon 6	g.8755T > C	c.746T > C	p.Ile249Thr	Mature	Donnarumma, et al., 2007
Exon 6	g.8758A > G	c.749A > G	p.Asp250Gly	Mature	Donnarumma, et al., 2007
Exon 7	g.9109C > T	c.830C > T	p.Ala277Val	Mature	Gelb, et al., 1998
Exon 7	g.9109C > A	c.830C > A	p.Ala277Glu	Mature	Hou, et al., 1999
Exon 7	g.9171T > C	c.892T > C	p.Trp298Arg	Mature	Nishi, et al., 1999
Exon 8	g.9186G > A	c.908G > A	p.Gly303Glu	Mature	Toral-Lopez et al., 2010
Exon 8	g.11474T > C	c.926T > C	p.Leu309Pro	Mature	Haagerup, et al., 2000
Exon 8	g.11479G > C	c.931G > C	p.Ala311Pro	Mature	Nishi, et al., 1999
Exon 8	g.11482C > G	c.934C > G	p.Arg312Gly	Mature	Hou, et al., 1999
Exon 8	g.11501G > A	c.953G > A	p.Cys318Tyr	Mature	Bertola et al., 2010
Exon 8	g.11503G > T	c.955G > T	p.Gly319Cys	Mature	Donnarumma, et al., 2007
**Nonsense**					
Exon 3	g.2146A > T	c.154A > T	p.Lys52X	Pro	Hou, et al., 1999
Exon 5	g.4266C > T	c.568C > T	p.Gln190X	Mature	Hou, et al., 1999
Exon 6	g.8730C > T	c.721C > T	p.Arg241X	Mature	Gelb, et al., 1996
**Frameshifts (duplication)**					
Exon 2	g.1591-1592dupGA	c.60_61dupGA	p.Ile21ArgfsX29	Pro	Donnarumma, et al., 2007
Exon 4	g.2359dupA	c.282dupA	p.Val95SerfsX9	Pro	Donnarumma, et al., 2007
**Frameshifts (deletion)**					
Exon 3	g.2230delG	c.238delG	p.Asp80ThrfsX2	Pro	Fratzl-Zelman, et al., 2004
Exon 5	g.4124delT	c.426delT	p.Phe142LeufsX19	Mature	Fujita, et al., 2000
**Splicing**					
Intron2	g.2112G > A	c.121-1G > A	p.del41Val-81Met	Pro	Haagerup, et al., 2000
Exon 7	g.9169G > A	c.890G > A; 785_890del	p.Gly262AlafsX70	Mature	Donnarumma, et al., 2007
**Stop codon**					
Exon 8	g.11538A > G	c.990A > G	p.X330TrpextX19	Mature	Gelb, et al., 1996

The reported families and characteristics of different mutations are summarized in Table 2. In addition to paternal uniparental disomy in 1 family, compound heterozygous mutations were found in 14 afflicted families (23.73%), while homozygous mutations were found in 44 afflicted families (74.58%). Of the 59 unrelated families, 37.29% were from Europe while 30.51% came from Asia.

**Table 2:** Genotypes of pycnodysostosis patients from different nationalities

Allele 1	Allele 2	Patients reported	Unrelated families	Nationality of patients	Reference
c.436G > C	c.436G > C	1	1	Algerian	Osimani, et al., 2009
c.436G > C	c.721C > T	1	1	American Hispanic	Gelb, et al., 1996
c.235G > A	c.238delG	1	1	Austria	Fratzl-Zelman, et al., 2004
c.934C > G	c.934C > G	1	1	Austria	Fratzl-Zelman, et al., 2004
c.830C > T	c.830C > T	1	1	Belgian	Gelb, et al., 1998
c.953G > A	c.953G > A	2	2	Brazil	Bertola et al., 2010
c.953G > A	c.721C > T	1	1	Brazil	Bertola et al., 2010
c.721C > T	c.436G > C	1	1	Brazil	Bertola et al., 2010
c.721C > T	c.721C > T	2	2	Brazil	Bertola et al., 2010
c.494A > G	c.721C > T	1	1	Caucasian	Laffranchi et al., 2010
c.154A > T	c.236G > A	2	1	Caucasian	Ho, et al., 1999
c.560A > C	c.560A > C	1	1	Chinese	Li, et al., 2009
c.121-1G > A	c.926T > C	1	1	Denmark	Haagerup, et al., 2000
c.236G > A	c.926T > C	1	1	Denmark	Haagerup, et al., 2000
c.926T > C	c.926T > C	6	3	Denmark	Haagerup, et al., 2000
c.890G > A; 785_890del	c.890G > A; 785_890del	1	1	Egypt	Donnarumma, et al., 2007
c.136C > T	c.136C > T	3	1	Germany	Schilling, et al., 2007
c.934C > G	c.934C > G	2	1	Honduran	Hou, et al., 1999
c.830C > A	c.830C > A	1	1	Indian	Hou, et al., 1999
c.990A > G	c.990A > G	16	1	Israeli Arab	Gelb, et al., 1996
c.20T > C	c.580G > A	1	1	Italy	Donnarumma, et al., 2007
c.494A > G	c.721C > T	1	1	Italy	Donnarumma, et al., 2007
c.26T > C	c.26T > C	2	2	Japanese	Nishi, et al., 1999; Fujita, et al., 2000
c.26T > C	c.892T > C	1	1	Japanese	Nishi, et al., 1999
c.426delT	c.426delT	2	2	Japanese	Fujita, et al., 2000
c.830C > T	c.830C > T	3	3	Japanese	Nishi, et al., 1999; Fujita, et al., 2000
c.721C > T	c.721C > T	10	1	Mexican	Johnson, et al., 1996
c.908G > A	c.908G > A	3	1	Mexican	Toral-Lopez et al., 2010
c.60_61dupGA	c.60_61dupGA	3	1	Moroccan	Donnarumma, et al., 2007
c.436G > C	c.436G > C	1	1	Moroccan	Rothenbuhler et al., 2010
c.436G > C	c.436G > C	2	1	Moroccan Arab	Gelb, et al., 1996
c.282dupA	c.282dupA	1	1	Pakistan	Donnarumma, et al., 2007
c.728G > A	c.728G > A	5	1	Pakistani	Khan et al., 2010
c.749A > G	c.749A > G	1	1	Pakistan	Donnarumma, et al., 2007
c.830C > T	c.830C > T	5	3	Pakistan	Donnarumma, et al., 2007; Naeem, et al., 2009
c.955G > T	c.955G > T	1	1	Pakistan	Donnarumma, et al., 2007
c.721C > T	c.721C > T	2	2	Portuguese	Hou, et al., 1999; Donnarumma, et al., 2007
c.830C > A	c.830C > A	1	1	Portuguese	Hou, et al., 1999
c.635A > G	c.721C > T	1	1	Spanish	Hou, et al., 1999
c.721C > T	c.746T > C	1	1	Spanish	Donnarumma, et al., 2007
c.721C > T	c.721C > T	1	1	Spanish	Rothenbuhler et al., 2010
c.931G > C	c.931G > C	2	1	Swiss	Nishi, et al., 1999
c.436G > C	c.436G > C	1	1	Tunisia	Donnarumma, et al., 2007
c.154A > T	c.236G > A	1	1	*	Hou, et al., 1999
c.568C > T	c.568C > T	1	1	*	Hou, et al., 1999
c.830C > T	c.830C > T	1	1	*	Hou, et al., 1999
c.422C > T	c.422C > T	1	1	Unknown	Chavassieux, et al., 2008
c.721C > T	c.721C > T	3	1	Unknown	Everts, et al., 2003

*: Two of the three families are from northern Europe, while the other is from Czech (no detailed record can be found in corresponding papers).

### Characteristics of mutant CTSK proteins

In order to determine CTSK expression, monocyte-derived macrophages were isolated from the peripheral blood of 2 siblings suffering from pycnodysostosis and their unaffected parents. Western blot revealed no detectable expression of either the proform or mature form of CTSK in either affected sibling with p.Gly79Glu and p.Lys52X. The levels of both proform and mature forms of CTSK in the father, a carrier of p.Gly79Glu, were nearly half that in normal controls, while the levels in the mother, a carrier of p.Lys52X, were more severely decreased [50]. In another study, monocytes were isolated from the peripheral blood of a patient with p.Ala141Val and induced to differentiate into osteoclasts *in vitro*. As a result of the mutation, the ability of the patient-derived cells to resorb bone was significantly decreased [46].

Functional properties of CTSK mutants (p.Leu7Pro, p.Leu9Pro, p.Gly79Glu, p.Gly146Arg, p.Gln165Arg, p.Gly194Ser, p.Tyr212Cys, p.Ile249Thr, p.Asp250Gly, p.Ala277Glu, p.Ala277Val, p.Arg312Gly, p.Gly319Cys, and p.X330Trp) were examined by transient expression in COS-7 cells, 293 cells, and *Pichia pastoris *GS115 cells, respectively [14,17,20,54]. Western blot analysis revealed that the mutants affecting residues of the mature domain yielded a mature form of a nonfunctional protein, while the mutants p.Leu7Pro, p.Leu9Pro, and p.X330Trp yielded a trace amount of this protein. In order to further understand the protein consequences of these missense mutations, amino acid changes of the mutant proteins, including p.Leu7Pro, p.Leu9Pro, p.Gln165Arg, p.Gly194Ser, p.Ile249Thr, p.Asp250Gly, and p.Gly319Cys, were modeled into the three-dimensional structure of the full-length CTSK. These mutations are predicted to affect the conformation of the protein [14].

All of these methods, including isolation of monocytes from the pycnodysostosis patients and transfection in COS-7 cells, 293 cells, and *P. pastoris *GS115 cells, demonstrated that CTSK mutants are functionally different from the wild type.

### *Ctsk*^-/- ^mouse models

The murine *Ctsk *gene maps to chromosome 3, and its predicted amino acid sequence is highly homologous to the human protein (85% identity; 93% similarity) [11]. *Ctsk*^-/- ^mouse models play quite an important role in studying the nature and function of *Ctsk *in osteoclasts and other cells, in detecting the mechanisms of phenotypes of pycnodysostosis, and even in optimizing therapeutic strategies (including gene therapy) for the treatment of this genetic disorder [34,55]. A homozygous null mutation in the mouse *Ctsk *gene was first established in 1998. *Ctsk*^-/- ^mouse strains have been generated in different genetic backgrounds since then [34,37,56-58]. All *Ctsk*^-/- ^mouse strains could mimic the phenotype of human pycnodysostosis to different extents.

Generally, *Ctsk *deficient mice may survive and are fertile. The phenotype of *Ctsk*^*-/- *^mice resembles clinical characteristics of the human pycnodysostosis in several aspects, such as the presence of osteopetrosis, reduced bone marrow cellularity, and splenomegaly after 2 months of age [37,58]. Using radiography, micro-computed tomography, and histological analyses, *Ctsk*^-/- ^mice were shown to display an osteopetrotic phenotype with excessive trabeculation of the bone marrow space [56]. Deficiency of *Ctsk *affects the late stage of the osteoclastic resorption cycle. As a result, *Ctsk*^-/- ^mice are unique among the currently available osteopetrotic mouse models [56].

*Ctsk*^-/- ^mice generally have minor craniofacial anomalies, such as increased density of the maxilla and paranasal sinus bones as well as alterations in mandibular shape [37]. Other skeletal changes seen in pycnodysostosis patient, such as growth retardation, phalangeal deformities, and delayed suture closure in the skull, have seldom been reported in *Ctsk*^-/- ^mice. Recent studies found that the pycnodysostosis phenotype in *Ctsk*^-/- ^mice is background-dependent. Compared with other strains of *Ctsk*^*-/- *^mice, the phenotypical characteristics of 129/Sv *Ctsk*^*-/- *^mice were similar to those of human pycnodysostosis, including short stature, osteopetrosis in the long bones, spondylolysis, acroosteolysis, bone fragility, separated cranial sutures with open fontanels, loss of the mandibular angle, lack of normal occlusion, and enhanced open bite [34].

A transgenic mouse model overexpressing the *Ctsk *gene showed that excess *Ctsk *production resulted in a high turnover of the metaphyseal trabecular bone. Enhanced bone resorption in these mice led to increased osteoblast numbers and activities, possibly mediated by core-binding protein α1, a transcription factor essential for osteoblast differentiation [56].

### Clinical relevance

A series of typical features in clinical and radiological examinations have been observed in pycnodysostosis. We summarized the manifestations in 97 reported cases (Table 3) [1-10,17,35,42-47,50,52,53,59-91]. The most common phenotype of pycnodysostosis is short stature, which was reported in 95.9% of the 97 reported cases. The next most common phenotype is an increase in bone density, which was reported in 88.7% of the 97 patients. Open fontanels and sutures with frontal and parietal bossing, frequent fractures, hypoplasia of the maxilla and mandible with an obtuse mandibular angle, and stubby hands and feet with acroosteolysis of the distal phalanges were identified in more than 50% of the pycnodysostosis patients. Approximately one-third of the pycnodysostosis patients showed prominent eyes with bluish sclera. Additionally, these patients also show some dental defects, such as delayed eruption of permanent teeth with persistence of deciduous teeth, dental crowding, and malocclusion, which may be ignored by clinicians.

**Table 3:** Typical clinical features of pycnodysostosis*

Typical features	Positive	Negative	Not mentioned
Short stature (<150 cm)	93(95.9%)	0	4(4.1%)
Increase of bone density	86(88.7%)	0	11(11.3%)
Open fontanels and sutures	68-73(70.1%-75.3%)	0	24-29(24.7%-29.9%)
Frontal and parietal bossing	67-72(69.1%-74.2%)	0	25-30(25.8%-30.9%)
Fractures	65(67.0%)	3(3.1%)	29(29.9%)
Obtuse mandibular angle	63(64.9%)	0	34(35.1%)
Hypoplasia of the jaws	53-61(54.6%-62.9%)	0	36-44(37.1%-45.4%)
Stubby hands and feet with osteolysis of the distal phalanges	43-50(44.3%-51.5%)	0	47-54(48.5%-55.7%)
Prominent eyes with bluish sclerae	34(35.1%)	0	63(64.9%)
Grooved palate	20-28(20.6%-28.9%)	0	69-77(71.1%-79.4%)
Dysplastic nails	26(26.8%)	0	71(73.2%)
Clavicular dysplasia	24(24.7%)	0	73(75.3%)
Nonpneumatised paranasal sinuses	17(17.5%)	0	80(82.5%)
Beaked nose	16(16.5%)	0	81(83.5%)

*n = 97 (44 females, 42 males, and 11 unknown)

In addition to the typical manifestations mentioned above, some unusual findings, including hearing loss [5], central giant-cell granuloma of the maxilla [82], congenital pseudarthrosis of the clavicle [7], spondylolysis [79], and bone marrow hypoplasia with compensatory splenomegaly [76], were reported in pycnodysostosis patients.

Pycnodysostosis patients usually have normal life expectancies and mentations. Results of laboratory investigations, including leukocyte and thrombocyte number; mean corpuscular volume (MCV); and the levels of hemoglobin (Hb), plasma phosphate, calcium, and alkaline phosphatase, are usually within normal limits [1,3-5].

### Physiopathological mechanism of pycnodysostosis

Abnormal bone metabolism is the typical physiopathological characterics of pycnodysostosis. The most common phenotype of pycnodysostosis is short stature. Based on the results of animal experiments, the short stature in pycnodysostosis may be related to the reduced size of the long bones [37].

In addition, pycnodysostosis patients usually suffer from pathologic fractures as a result of brittle, chalk-like bones. Histomorphometric and biomechanical assays in *Ctsk*^-/- ^mice have suggested that CTSK may play a critical role in matrix formation as well as breakdown. Large amounts of brittle, poorly organized matrix were formed in the absence of *Ctsk *gene, which corresponds to the bone fragility observed in patients with *CTSK *deficiency [57].

The coexistence of increased bone density in long bones (osteosclerosis) and osteolysis in the distal phalanges and calvariae is a typical characteristic of pycnodysostosis with *CTSK *mutation. One explanation may be the site-specific variations in bone homeostasis. It was reported that CTSK is clearly important to bone resorption in rapidly remodeling bones (*e.g.*, the long bones), while it is not the most essential factor in regulating the bone resorption in bones with low turnover rates, such as the calvaria and epiphysis [34,37,48]. On the other hand, animal experiments have shown that metalloproteinases participate in osteoclastic resorption of calvarial bones, but not of long bones [34]. The increased number of *Ctsk*^-/- ^osteoclasts may result in excessive bone resorption in the distal end of long bones (osteolysis), and the impaired ability of osteoclasts may account for the osteopetrosis in the main body of long bones [34,37,48,56].

Immunophenotype analysis of the cell types in the bone marrow of *Ctsk*^-/- ^mice revealed reductions in absolute cell numbers, but not in the percentages of all the different subtypes, indicating that marrow hypoplasia results from the reduction in bone marrow cavity space, which is caused by osteopetrosis. As a result, extramedullary hematopoiesis occurs in the spleen (enlarged with a significantly elevated cellularity) to compensate for the reduced bone marrow cellularity [37].

### Diagnosis and differential diagnoses

Although *CTSK *gene mutation analysis is the confirmatory test for pycnodysostosis, the diagnosis can be established primarily based on the aforementioned clinical features and radiograph findings [5]. On the other hand, pycnodysostosis should be distinguished from other genetic bone diseases, particularly cleidocranial dysostosis (CCD) and osteopetrosis [5,47,68]. Clavicular hypoplasia and typical craniofacial characteristics, especially the open fontanels and cranial sutures in pycnodysostosis, may lead to misdiagnosis of cleidocranial dysplasia. However, increased bone density with recurrent fractures is highly suggestive of pycnodysostosis [92-95]. Short stature and generalized osteosclerosis with multiple fractures in pycnodysostosis patients has been misdiagnosed as osteopetrosis [71,96-98]. However, acroosteolysis of the distal phalanges and unclosed cranial sutures and fontanels are indicative of pycnodysostosis.

### Treatment

To date, no specific treatment has been validated in pycnodysostosis with the exception of symptomatic management [5,46]. Because bone fractures are a primary threat to these patients, it is important to prevent fracture-causing factors. These patients are likely to visit dentists because oral deformities severely affect their life. Here, we would like to emphasize the role of dentists in the diagnosis and treatment of this condition. Maintenance of oral hygiene and regular dental care may help prevent some oral complications. However, the greater bone density increases the probability of developing postextraction osteomyelitis. Thus, risk factors should be carefully addressed while planning tooth extraction and other treatments. In summary, it is quite important to establish a correct diagnosis as early as possible to prevent fractures and ensure a better quality of life [5,6,54].

### Future prospects

Genotype-phenotype correlation draws great attention in the field of genetic disorder research. Clinical features with *CTSK *genetic analysis were reported in 42 pycnodysostosis patients from 24 families [1,3,8,9,17,42-47,50,52,53]. However, the detailed clinical information was poorly described in most of the reports with molecular data. Lack of good clinical information makes it difficult to evaluate of the genotype-phenotype correlation of pycnodysostosis. More detailed information about the clinical and genetic characteristics in pycnodysostosis patients are necessary for the further research to elucidate the CTSK genotype-phenotype correlation.

Research on specific approaches to correct the abnormal bone metabolism in pycnodysostosis is another hot topic. Due to providing normal osteoclasts and osteoclast-targeted enzymes, bone marrow transplantation is drawing the increasing attention. Gene replacement strategies are other alternative choices. However, considerable research is required in this area [54].

Recently, CTSK was shown to play an important role in autoimmune and inflammatory diseases by animal and *in vitro *experiments [26]. If the role of CTSK in the human immune system is confirmed [99], it will be helpful in further understanding of the mechanism of pycnodysostosis and in designing specific treatment strategies.

## List of abbreviations

COS-7 cells: COS-7 SV40-transformed kidney cells; CTSK, *Ctsk*: cathepsin K; Hb: hemoglobin; IL-6: interleukin 6; IL-23: interleukin 23; MCV: mean corpuscular volume; RANKL: receptor activator of nuclear factor kappa B ligand; Th17: T helper 17 cells

## Competing interests

The authors declare that they have no competing interests.

## Authors' contributions

YX participated in reviewing papers and drafted the manuscript. TC participated in its design and helped to modify the manuscript. SS participated in its design and helped to modify the manuscript. WW participated in reviewing papers and analyzing data. YZ participated in finding papers and analyzing data. TM conceived of the study, and participated in its design and coordination and helped to modify the manuscript. XD conceived of the study, and participated in its design and coordination and helped to draft the manuscript. All authors read and approved the final manuscript.
